# Choroïdite serpigineuse maculaire

**DOI:** 10.11604/pamj.2015.21.150.7188

**Published:** 2015-06-24

**Authors:** Meriem Abdellaoui, Hicham Tahri

**Affiliations:** 1 Faculté de Médecine et de Pharmacie de Fès, Service d'Ophtalmologie, CHU Hassan II, Fès, Maroc

**Keywords:** Uveite, Choroidite, macula, Uveitis, choroiditis, macula

## Image en medicine

**Figure 1:**
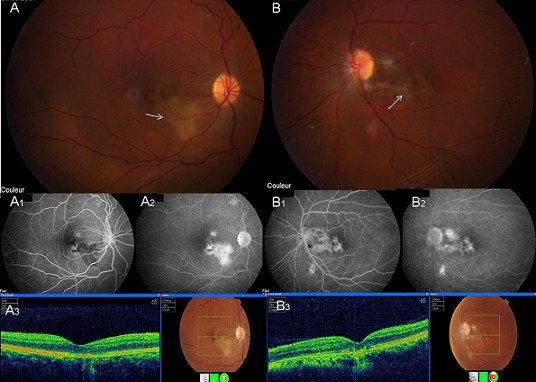
(A, B): Photographie du fond d’œil montrant une lésion maculaire bilatérale profonde à bordure serpigineuse de couleur jaunâtre (flèches blanches) sans anomalies vasculaires rétiniennes associées; A1 et A2: l'angiographie à la fluorescéine montre à droite, une hypofluorescence précoce des lésions rétiniennes de forme serpigineuse avec imprégnation tardive; B1 et B2: l'angiographie à la fluorescéine montre à gauche un effet fenêtre des lésions rétiniennes avec diffusion tardive minime;A3 et B3: l'OCT maculaire objective un amincissement du tissu rétinien avec irrégularité et hyper réflectivité du complexe épithélium pigmentaire-choriocapillaire et discontinuité de la ligne de jonction segment interne-segment externe des photorécepteurs plus marquées à gauche qu’à droite

Mlle C.B, 37 ans, avec antécédent de malvoyance de l’œil gauche depuis l'enfance, admise dans notre service pour baisse visuelle droite depuis 15 jours. L'acuité visuelle corrigée est de 5/10 P4 à droite, limitée à 1/10 P14 à gauche avec anisomyopie. Le segment antérieur est normal, le vitré est clair au niveau des deux yeux. Le fond d’œil montre une lésion maculaire bilatérale profonde à bordure serpigineuse de couleur jaunâtre sans anomalies vasculaires rétiniennes associées (A et B). L'angiographie à la fluorescéine montre à droite, une hypofluorescence précoce des lésions avec imprégnation tardive (A1 et A2), tandis qu’à gauche il y a plutôt un effet fenêtre avec diffusion tardive minime (B1 et B2). L'OCT maculaire objective un amincissement du tissu rétinien avec irrégularité et hyper réflectivité du complexe épithélium pigmentaire-choriocapillaire et discontinuité de la ligne des photorécepteurs des deux yeux (A3 et B3). Le bilan étiologique clinique et paraclinique y compris les sérologies virale, toxoplasmique et l'intradermoréaction à la tuberculine, est revenu négatif. Le diagnostic de choroïdite serpigineuse maculaire bilatéral active à droite et séquellaire à gauche, est retenu. La patiente a reçu un bolus de méthylprédnisolone (10mg/kg/J) pendant trois jours. L’évolution, après 4mois de suivi, est marquée par l'amélioration de l'acuité visuelle à droite qui est passée à 10/10 corrigée avec cicatrisation de la lésion maculaire, cependant une autre lésion suprapapillaire droite est apparue. Figure 1

